# Application of moving particle semi-implicit (MPS) method on retro-oil fluid using three-dimensional vitreous cavity models from magnetic resonance imaging

**DOI:** 10.1038/s41598-022-05886-5

**Published:** 2022-02-02

**Authors:** Makoto Gozawa, Naoki Watanabe, Kentaro Iwasaki, Yoshihiro Takamura, Masaru Inatani

**Affiliations:** 1 Department of Ophthalmology, Faculty of Medical Sciences, University of Fukui, 23-3 Shimoaizuki, Matsuoka, Eiheiji, Yoshida, Fukui 910-1193 Japan; 2 Prometech Software Inc, Round Terrace Fushimi, 3F, 17-26, Nishiki 1-chome, Naka-ku, Nagoya 460-0003 Japan

**Keywords:** Diseases, Eye diseases, Retinal diseases

## Abstract

Silicone oil (SO) is a safe and widely used intraocular tamponade agent for treating complicated vitreoretinal diseases, such as retinal detachments (RRDs) with inferior proliferative vitreoretinopathy (PVR). However, as the human vitreous cavity is irregularly shaped, it is difficult to predict the area of the inferior retina covered with SO and the retro-oil fluid currents in each patient. Here, we performed fluid simulation analysis using the moving particle semi-implicit method on the oil cover rates and absolute velocity gradient of retro-oil fluid to the retina using vitreous cavity models derived from magnetic resonance imaging of patients to determine the appropriate amount of SO and postoperative position to achieve a sufficient tamponade effect on the inferior retina. In all seven vitreous cavity models tested, the inferior quadrant of the retina was completely covered by SO in more positions and the absolute velocity gradient of the retro-oil fluid in contact with the retinal wall caused by eye and head movements was lower when the vitreous cavity was filled with 95% SO and 5% retro-oil fluid versus 80% SO and 20% retro-oil fluid. Taken together, these findings have clinical implications for the treatment of complicated RRDs with inferior PVR requiring SO tamponade.

## Introduction

Silicone oil (SO) is a safe and well-tolerated intraocular tamponade agent widely used for the treatment of complicated vitreoretinal diseases such as inferior rhegmatogenous retinal detachments (RRDs), proliferative vitreoretinopathy (PVR), giant retinal tears, traumatic retinal detachments, and some cases of tractional retinal detachments^1^. As SO is less dense than water, SO has positive buoyancy and floats, and the contact angle is smaller than that of gas to the retina^2^. Therefore, the tamponade efficiency of SO to the inferior quadrant of the retina decreases compared with that of SO to other quadrants. Furthermore, the actual shape of the human vitreous cavity is not perfectly spherical, especially in myopic eyes^3^. Therefore, the percentage of the vitreous cavity that should be filled with SO in individual cases and the postoperative positional restrictions necessary to achieve sufficient coverage by SO in the inferior retina are difficult to predict. These are some of the reasons why a 100% single-surgery anatomical success rate for complicated retinal detachment with inferior proliferative vitreoretinopathy has not been reached yet^4^.

Intraocular fluid currents caused by saccadic eye and head movements exert shear stress on the retina, and this stress has been considered as a significant pathogenic mechanism that causes retinal detachment^5^. Angunawela et al. evaluated fluid shear stress on the retinal wall after vitrectomy with gas tamponade using computational eye model. They reported that sudden head movements may cause shear stresses that may exceed the strength of retinal adhesion^5^. In SO-filled eyes, intraocular fluid called as “retro-oil fluid” accumulates in the space between the SO and the retina^6,7^. Although saccadic eye and head movements may cause retro-oil fluid currents, no reports have described shear stresses on the retinal wall caused by retro-oil fluid currents.

Computational fluid dynamics (CFD) analysis is a powerful tool for evaluating the distribution of gas and fluid that considers the effects of physical properties such as contact angles and surface tension. Angunawela et al. reported intraocular fluid shear stresses on the retinal wall using CFD software^5^. Previously, we reported the appropriate postoperative positions after pars plana vitrectomy (PPV) with gas tamponade for RRDs using CFD software and the traditional mesh-based method^8^. Although the mesh-based method is easy to perform if the analysis domain has a simple shape, such as the spherical domain used in previous reports, many steps are required to create the mesh if the domain has an irregular or complex shape, and the calculations sometimes terminate abnormally.

Particle methods include meshfree methods for simulation of nonlinear free surface flow and flow with large deformable interfaces in complicated shapes, such as the human vitreous cavity—especially myopic eyes. Compared to traditional mesh-based methods, these particle methods enable easier analysis of moving interfaces and free surfaces. The moving particle semi-implicit (MPS) method was introduced by Koshizuka and Oka to simulate fragmentation of incompressible fluids^9^. Particle methods have been widely applied in engineering^10–12^ and in some medical studies, such as models of swallowing and blood flow^13,14^. However, no previous report has analyzed intraocular fluid currents using particle methods and the vitreous cavity shapes of real patients.

This study aimed to use the MPS method to perform fluid simulation analysis using SO-filled 3-D vitreous cavity models obtained from magnetic resonance imaging (MRI) of real patients to determine the appropriate amount of SO and postoperative position to achieve a sufficient tamponade effect on the inferior retina.

## Methods

### Patient selection

This study was approved by the institutional review board of the University of Fukui Hospital, Fukui, Japan, and adhered to the tenets of the Declaration of Helsinki. Written informed consent was obtained from all patients after detailed explanation of the procedures involved. Patients aged ≥ 20 years who underwent orbital MRI at the Fukui University Hospital between December 1, 2014, and March 31, 2016, were included. Patients with a history that could affect the shape of the vitreous cavity at the time of MRI, such as scleral buckling, ocular trauma, vitreous hemorrhage, and ocular tumor, were excluded.

### MRI

High-resolution orbital images were obtained using a 3-Tesla scanner (Discovery MR 750 3.0T; GE Healthcare, Milwaukee, WI) in combination with a 32-element phased-array head coil with fast imaging sequences using steady-state acquisition cycled phases. The imaging parameters were as follows: repetition time, 5.6 ms; echo time, 2.7 ms; field of view, 180 mm; and matrix size, 320 × 288; and slice thickness, 0.6 mm with an overlap thickness perpendicular to the plane containing both optic nerves. The subjects were repeatedly asked to avoid unnecessary movements during scanning.

### Creating and designing the 3-D vitreous cavity model from MRI scans

**Figure 1:**
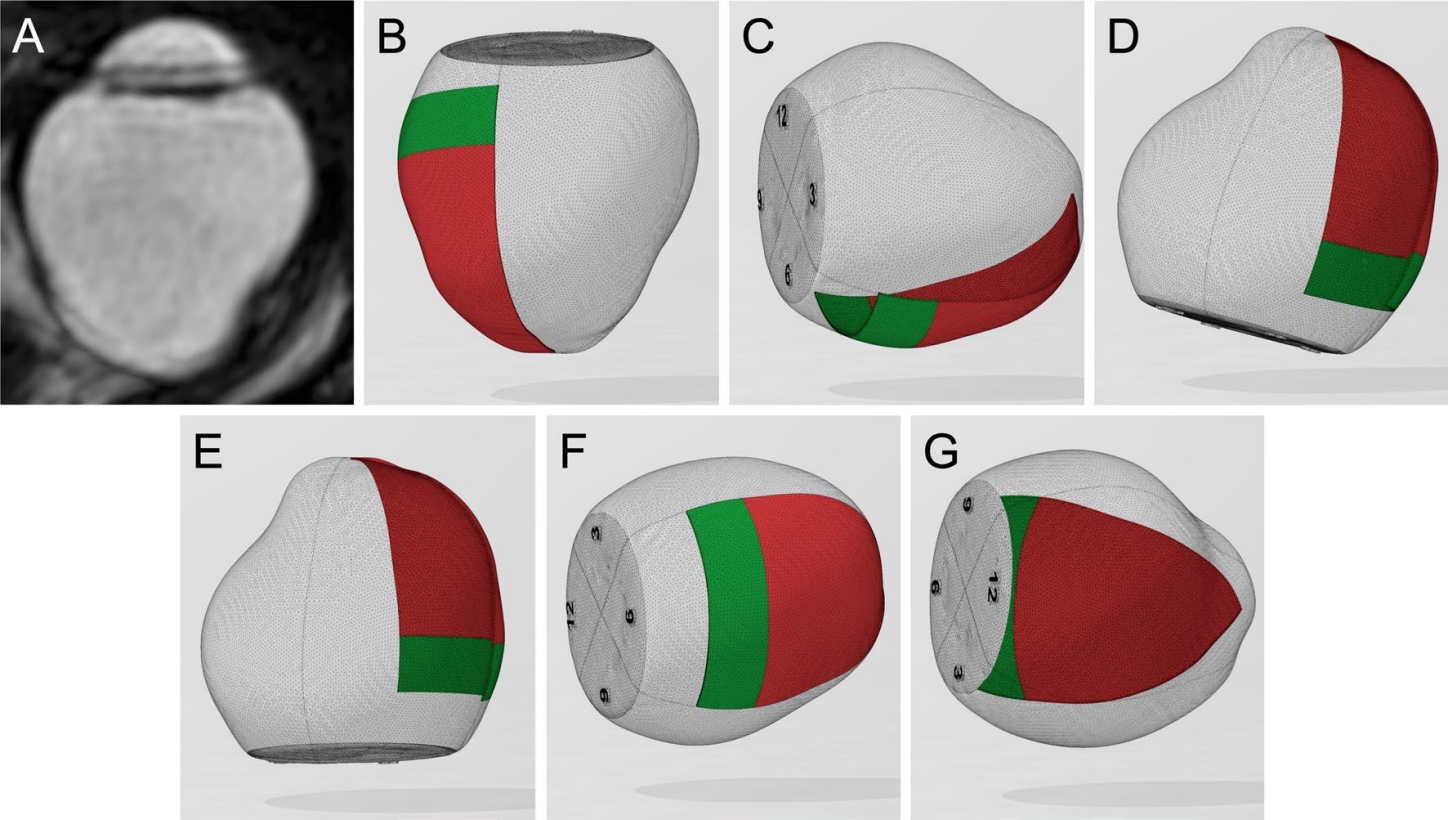
Directions of the three-dimensional vitreous cavity in case 1. (**A**) The real MRI image, (**B**) Supine, (**C**) sitting, (**D**) prone with closed eyes, (**E**) prone, (**F**) lower temporal, and (**G**) lower nasal positions. The inferior-anterior and inferior-posterior retina are colored green and red.

First, we extracted the 3-D vitreous cavity model from the MRI scans in all the cases as the standard triangulated language format and measured the vitreous volume using Expert INTAGE (Cybernet systems Co., Ltd., Tokyo, Japan). Next, we defined the intersection of the perpendicular line from the angle with the ocular surface as the surgical limbus on MRI scans. Then, the 3-D vitreous cavity model was imported and designed using three-dimensional (3-D) modeling software (3D Builder; Microsoft Corporation, WA, USA). First, the 3-D vitreous cavity was hollowed out to generate particles inside while maintaining the original shape of the vitreous cavity. Next, we separated the 3-D vitreous cavity at 1:30, 4:30, 7:30, and 10:30 into four quadrants and then defined the position of the ora serrata of the inferior retina as the anatomical differences as 6.7 mm posterior to the limbus. Then, we defined the equator as 6.5 mm posterior to the ora serrata in the inferior retinal area, according to the vortex vein ampullae^15,16^. Therefore, the inferior quadrant of the retina was separated into two parts, inferior-anterior and inferior-posterior (Fig. 1).

### CFD analysis

#### Relevant equations and solution methodology

Particleworks 7.1 (Prometech Software Inc., Tokyo, Japan) was used as a solver to simulate the currents of SO and retro-oil fluid using the MPS method. The fundamental governing equations of the MPS method are a continuum equation (mass conservation law) and Navier–Stokes equations (momentum conservation law) written as follows:

1 $$ \begin{array}{*{20}c} {\frac{{D\vec{u}}}{Dt} = 0} \\ \end{array} $$

2 $$ \begin{array}{*{20}c} {\frac{{D\vec{u}}}{Dt} = - \frac{1}{\rho }\nabla P + \upsilon \nabla^{2} \vec{u} + \vec{g}} \\ \end{array} $$

where $\rho$ is density, $\vec{u}$ is velocity, $P$ is pressure, $\upsilon$ is the kinematic viscosity coefficient, and $\vec{g}$ is gravity acceleration. The acceleration added to the surface was calculated from the fluid surface curvature as follows:

3 $$ \begin{array}{*{20}c} {\frac{{D\vec{u}}}{Dt} = - \frac{1}{\rho }\nabla P + \upsilon \nabla^{2} \vec{u} + \vec{g} + \frac{1}{\rho }\sigma \kappa \delta \vec{n}} \\ \end{array} $$

where $\sigma$ is the surface tension coefficient, $\kappa$ is the curvature, $\delta$ is a delta function to ensure the surface tension works only on the surface, and $\vec{n}$ is a unit vector in the vertical direction to the surface. In addition, wettability can be controlled by changing the contact angle between the fluids and walls.

### Boundary condition

The wall slip condition was no-slip. The MPS method does not use a wall as a calculation point; therefore, the no-slip condition is expressed by the viscosity between the fluid and wall as follows:

4 $$ \begin{array}{*{20}c} {\upsilon_{fs} = \upsilon_{f} } \\ \end{array} $$

where $\upsilon_{fs}$ is the kinematic viscosity coefficient between the fluid and the wall and $\upsilon_{f}$ is the kinematic viscosity coefficient of the fluid.

The 3-D vitreous cavity of each case was imported and filled with combinations of 80% SO and 20% retro-oil fluid, and 95% SO and 5% retro-oil fluid. Particleworks 7.1 cannot measure shear stress but can analyze the absolute velocity gradient that is proportional to the shear stress in Newtonian fluids such as SO and water. Therefore, in this study, the absolute velocity gradient (1/s) caused by saccadic eye and head movements in a sitting position in which the inferior retina was at the lowest position was measured in each combination of SO and retro-oil fluid. The SO properties were as follows: temperature, 37 °C; density, 970 kg/m^3^; kinematic viscosity, 0.001 m^2^/s; and surface tension, 0.021 N/m. The retro-oil fluid properties were the same as those of water as follows: temperature, 37 °C; density, 1000 kg/m^3^; kinematic viscosity, 1e-6 m^2^/s; and surface tension, 0.072 N/m. The contact angle of the SO-retina interface was 18.2°. The setting values were as follows: particle size (initial distance between particles), 0.35 mm; gravity, − 9.8 m/s^2^.

### Definition of ocular movement parameters

Particleworks 7.1 (Prometech Software Inc., Tokyo, Japan) was used for examining the three types of saccadic eye movement^5^ in the sitting position.

1. Rotation around a vertical axis (horizontal saccadic movements)
2. Rotation around a horizontal axis (vertical saccadic movements)
3. Movement in the horizontal plane of the whole eye (rectilinear head movement)

The angular displacement (for rotation around the vertical and horizontal axes) was fixed at ⊿*θ* (dtheta) = 30°, whereas the maximum angular velocity was vtheta = 500°/s. We described the script for analysis by Particleworks 7.1 as follows:

Function derives value (*t*) {var dtheta = 30; var vtheta = 500; var t0 = 1.5 if (*t* < 2.0) returns dtheta/2 × (1 + math.tanh (2 × vtheta × (*t* − *t*0)/dtheta)); else returns dtheta;}.

We have applied the same values for both the horizontal and vertical saccades.

When the head moves, the eye has both components of rectilinear and angular acceleration. The contribution of rectilinear acceleration was examined separately by considering the case of an eye moving along a distance dX of approximately 0.1 m, with a maximum velocity of vX = 1 m/s, over a period of dX/vX of approximately 0.05 seconds^5^. To describe the rectilinear, unsteady movement of the eye caused by the movement of the head, we described the script for analysis by Particleworks 7.1 as follows:

Function get value (*t*) {var dX = 100; var vX = 1000; var *t*0 = 1.5 if (*t* < 2.0) return dX/2 × (1 + m.tanh (2 × vX × (*t* × *t*0)/dX)); else return dX;}.

### Direction of the 3-D vitreous cavity

Figure 1 shows the resting positions of the 3-D vitreous cavity, which represents the postoperative positions, and each position is reproduced by 3D Builder (Microsoft Corporation, WA, USA). We previously reported that even if patients superficially maintain a strict prone position with closed eyes, the mean supraduction angle (degrees) of the eyeball to the perpendicular line is positive (16.1°)^15^. Therefore, the six resting positions of the eyeball examined were as follows:

1. Supine: The direction of the eyeball is 90° upward from the horizontal line.
2. Sitting: The direction of the eyeball is parallel to the horizontal line.
3. Prone with closed eyes: The supraduction angle of the eyeball to the perpendicular line is 16.1°.
4. Prone: The direction of the eyeball is 90° downward from the horizontal line.
5. Lower temporal: The eyeball is rotated 90° from the sitting position such that the temporal retina was on the side of the horizon.
6. Lower nasal: The eyeball is rotated 90° from the sitting position such that the nasal retina is on the side of the horizon side.

### Measurement of oil cover rates of the inferior retina

**Figure 2:**
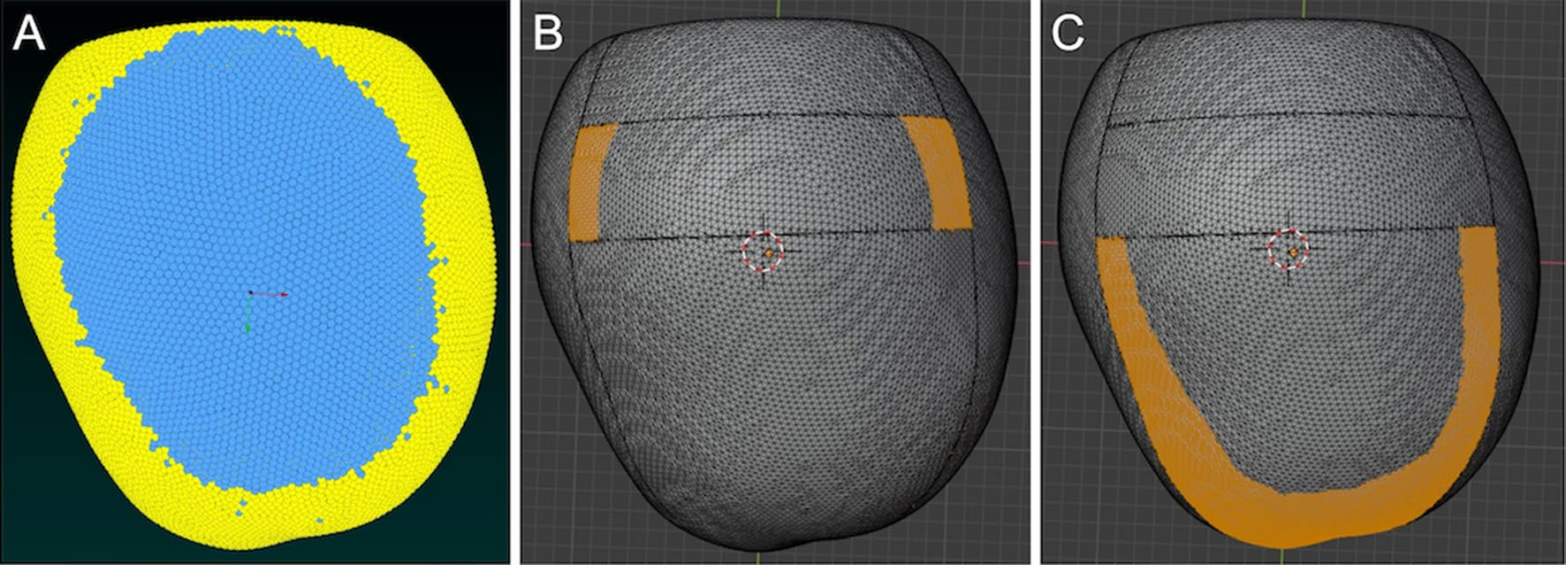
Measurement of the oil cover rate of the inferior retina in the sitting position with the vitreous cavity filled with 95% oil in case 1. (**A**) The three-dimensional vitreous cavity of case 1 filled with a combination of 95% silicone oil (yellow) and 5% retro-oil fluid (blue). (**B**) The parts of the inferior-anterior and (**C**) inferior-posterior retina that are in contact with the oil are shown in orange.

Blender 2.90 (https://www.blender.org/) was used for the measurement of oil cover rates (%) of the inferior-anterior and inferior-posterior retina (Fig. 2). The result of the CFD simulation analysis and the wire frame of the 3-D vitreous cavity are displayed simultaneously, and the oil covered area where the SO particles were contacting the retinal wall was measured. The oil cover rate of the inferior retina with 80% and 95% SO was calculated as (oil covered area)/ (total area of the inferior-anterior and inferior-posterior retina) × 100% in the supine, sitting, lower nasal, lower temporal, “prone with closed eyes,” and prone positions.

### Statistical analyses

Statistical analyses were performed using JMP 14 (SAS institute Inc., Tokyo, Japan). Tukey’s honestly significant difference test was used to compare the oil cover rates in each position in the oil 95% and 85% groups. The Wilcoxon signed-rank test was used to compare the oil cover rates at 95% and 80% oil in each position. P values < 0.05 were considered statistically significant.

## Results

### Patient characteristics

**Figure 3:**
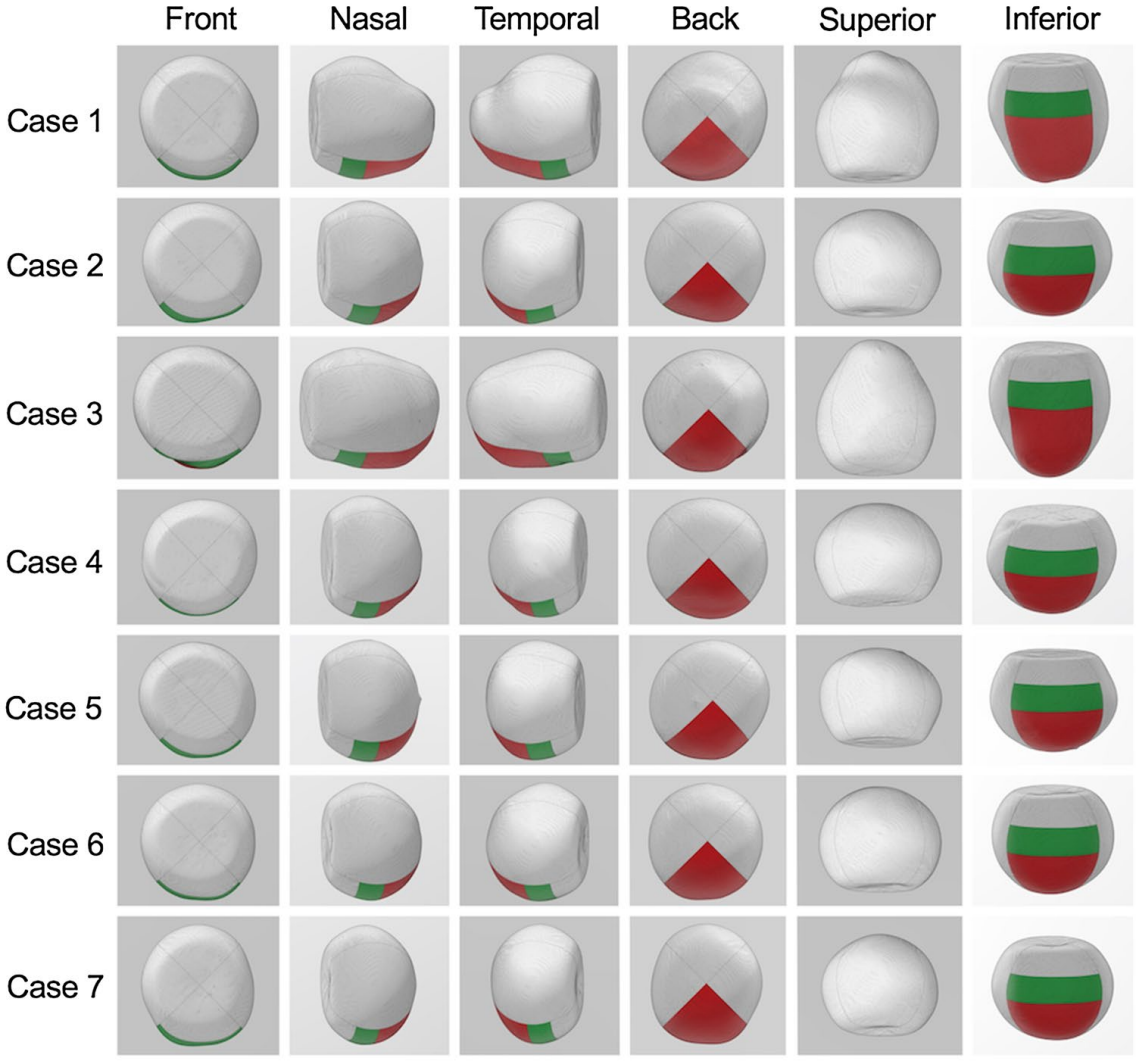
Three-dimensional vitreous cavities of all the cases. In all the cases, the inferior-anterior and inferior-posterior retina are painted green and red, respectively.

Seven pseudophakic eyes of seven patients (five men and two women) were included in the study. The mean patient age was 74.9 years (range, 62–85 years). The mean axial length was 26.79 mm (range, 23.53–33.14 years). The mean vitreous volume was 7.21 ml (range, 5.96–9.23 ml). Table 1 summarizes the patients’ characteristics. Figure 3 shows the shapes of the 3-D vitreous cavities made from the orbital MRI scans of all the cases using Expert INTAGE (Cybernet systems Co., Ltd., Tokyo, Japan).

**Table 1:** Patient characteristics.

Case	Age (years)	Sex	Eye	Lens status	Axial length (mm)	Vitreous volume (ml)
1	80	F	R	IOL	31.43	8.28
2	67	M	R	IOL	25.60	7.59
3	75	F	R	IOL	33.14	9.23
4	62	M	R	IOL	24.36	6.28
5	81	M	R	IOL	24.86	6.63
6	85	M	R	IOL	24.63	6.48
7	74	M	R	IOL	23.53	5.96

### Oil cover rates of the inferior-anterior retina

Table 2 shows the oil cover rates of the inferior-anterior retina in each position. When the vitreous cavity was 95% filled with SO, the oil cover rate was 100% except for the sitting position and was 34% in the sitting position. When the vitreous cavity was 80% filled with SO, the oil cover rate was 100% only in the supine and prone positions. In the sitting, lower nasal, lower temporal, and “prone with closed eyes” positions, the oil cover rates were 0%, 73%, 69%, and 39%, respectively.

**Table 2:** Mean oil contact rates of the inferior-anterior retina.

Silicone oil volume ratio to the vitreous cavity					
Position	95%	80%	*P*-value		
Supine	100%	100%			1
Sitting	34%	0%	< 0.0001*	< 0.0001**	0.0008^†^
Lower nasal	100%	73%		< 0.0001**	0.0008^†^
Lower temporal	100%	69%		< 0.0001**	0.0008^†^
Prone	100%	100%			1
Prone with closed eyes	100%	39%		< 0.0001**	0.0008^†^

*P*-value* within 95% group, *P*-value** within 80% group, *P*-value^†^ at 95% oil versus at 80% oil.

### Oil cover rates in the inferior-posterior retina

Table 3 shows the oil cover rates of the inferior-posterior retina in each position. When the vitreous cavity was 95% filled with SO, the oil cover rate was 100% except for the supine and sitting positions, and the oil cover rates were 78% and 62% in the supine and sitting positions, respectively. When the vitreous cavity was 80% filled with SO, the oil cover rate was 100% in the prone and “prone with closed eyes” positions. In the supine, sitting, lower nasal, and lower temporal positions, the oil cover rates were 59%, 25%, 87%, and 86%, respectively.

**Table 3:** Mean oil contact rates of the inferior-posterior retina.

Silicone oil volume ratio to the vitreous cavity					
Position	95%	80%	*P*-value		
Supine	78%	59%	< 0.0001*	< 0.0001**	0.0017^†^
Sitting	62%	25%	< 0.0001*	< 0.0001**	0.0022^†^
Lower nasal	100%	87%		< 0.0001**	0.0008^†^
Lower temporal	100%	86%		< 0.0001**	0.0008^†^
Prone	100%	100%			1
Prone with closed eyes	100%	100%			1

*P*-value* within 95% group, *P*-value** within 80% group, *P*-value^†^ at 95% oil versus at 80% oil.

### Absolute velocity gradient of the retro-oil fluid on the retinal wall

#### Horizontal saccadic eye movement

**Figure 4:**
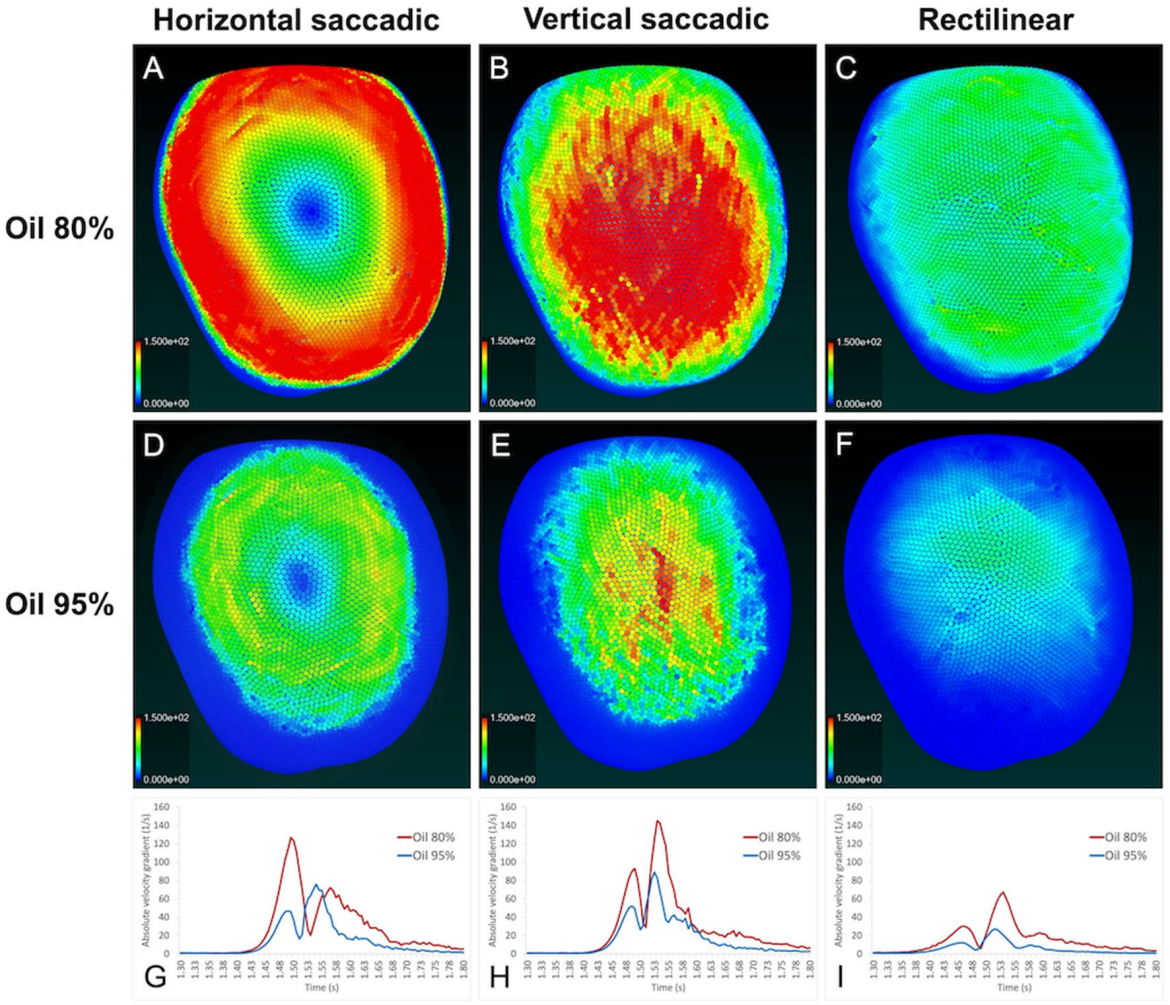
Absolute velocity gradient of the silicone oil and retro-oil fluid on the retinal wall in case 1. (**A**)–(**F**) Analysis images obtained at the maximum mean absolute velocity gradient. (**G**)–(**I**) Changes in the mean absolute velocity gradient caused by saccadic eye and rectilinear head movements.

Video 1 shows the horizontal saccadic eye movements of the 3-D vitreous cavity of case 1 filled with the combination of 80% SO and 20% retro-oil fluid as a representative case. In all the seven cases, the mean absolute velocity gradient of the retro-oil fluid contacting with the retinal wall increased bimodally in both 80% and 95% oil. The increase was concentric around the rotated axis. In 80% oil, the maximum value of the first increase of the mean absolute velocity gradient was higher than that of the second increase. On the other hand, in 95% oil, the maximum value of the second increase was higher than that of the first increase. In all the cases, the maximum value of the mean absolute velocity gradient in 80% oil was higher than that in 95% oil. (Fig. 4 and Supplementary files 1–6).

#### Vertical saccadic eye movement

Video 2 shows the vertical saccadic eye movements of the 3-D vitreous cavity of case 1 filled with the combination of 80% SO and 20% retro-oil fluid as a representative case. In all the cases, the mean absolute velocity gradient of the retro-oil fluid contacting with the retinal wall increased bimodally in both 80% and 95% oil, and the maximum value of the second increase was more than that of the first increase. In all the cases, the maximum value of the absolute velocity gradient of the retro-oil fluid contacting with the retinal wall in 80% oil was higher than in 95% oil both in the first and second increases. (Fig. 4 and Supplementary files 1–6).

### Rectilinear head movement

Video 3 shows the rectilinear head movements of the 3-D vitreous cavity of case 1 filled with the combination of 80% SO and 20% retro-oil fluid as a representative case. In all seven cases, the mean absolute velocity gradient of the retro-oil fluid contacting with the retinal wall increased bimodally in both 80% and 95% oil, and the maximum value of the second increase was more than that of the first increase. In all the cases, the maximum value of the absolute velocity gradient in 80% oil was higher than in 95% oil both in the first and second increases. In addition, the maximum value of the mean absolute velocity gradient was lower than those of the horizontal and vertical saccadic movements both in 80% and 95% oil (Fig. 4 and Supplementary files 1–6).

## Discussion

To the best of our knowledge, this is the first study to perform CFD simulation analysis using the MPS method for SO and retro-oil fluid in a 3-D vitreous cavity model from MRI scans of real patients. We demonstrated that the inferior retina was completely covered by SO in more positions when the vitreous cavity was filled with the combination of 95% SO and 5% retro-oil fluid than when the vitreous cavity was willed with the combination of 80% SO and 20% retro-oil fluid. The absolute velocity gradient of the retro-oil fluid in contact with the retinal wall caused by saccadic eye and head movements was lower when the vitreous cavity was filled with the combination of 95% SO and 5% retro-oil fluid than with 80% SO and 20% retro-oil fluid.

Although the traditional mesh-based method is easy to perform when the analysis domain has a simple shape^16–21^, meshing for irregular or complex shapes like the vitreous cavities of myopic eyes requires substantial manpower and mesh-collapsing issues may occur. In contrast, in the MPS method used here, the fluid itself is modeled as particles without a grid to represent space. Even if the analysis domain has a complex shape, the fluid can be easily modeled if computer-aided design data of the wall shape are available, and abnormal termination of the calculation due to mesh collapse can be avoided. In this study, despite the complex shapes of the vitreous cavities of real patients, the calculations were executed without any abnormal termination due to mesh-collapsing, including in cases 1 and 3 which had highly asymmetrical and irregularly shaped vitreous cavities. The MPS method greatly reduced the process of preparing the analytical model and thus allowed us to concentrate on the analysis. As MPS can be analyzed in a short time, The MPS method could be applied for selection of the tamponade agent or simulation of perioperative management based on the actual shape of the patient’s vitreous cavity.

SILIKON 1000 (Alcon Japan Ltd., Tokyo) is the only SO approved in Japan. Although the package inserts of SILIKON 1000 states that 80%–100% of the vitreous cavity should be filled with SO, no description is provided regarding which amount, between 80 and 100%, is better. In clinical practice, 100% exchange of the vitreous cavity with SO is difficult to achieve^4^. This is because the vitreous humor cannot be completely dissected, some residual subretinal fluid is absorbed postoperatively and the space is replaced by the aqueous humor, and the aqueous humor replaces the space created by the disappearance of residual gas in the eye during gas-to-oil replacement. In this study, we found that the inferior retina was not covered completely by the SO depending on the position even if the vitreous cavity was filled with 95% SO due to the existence of the contact angle and the meniscus. Therefore, for better coverage of the inferior retina with SO, injection of SO close to 100% in the vitreous cavity may be better and restricting the position depending on the location of breaks and percentage of oil to the vitreous cavity may be necessary. When the retinal breaks are in the inferior-anterior location, and medical, physical, or mental conditions prevent patients from maintaining the prone position, the supine position may be the appropriate position for covering all breaks.

We also found that the maximum value of the absolute velocity gradient of the retro-oil fluid contacting the retinal wall due to saccadic eye and head movements was higher for 80% oil than 95% oil. Since the viscosity of SO is greater than that of retro-oil fluid, SO follows the movement of the retinal wall more easily. In the 95% SO-filled eyes, there was more SO in contact with the retinal wall than in the 80% SO-filled eyes. This could mean that the flow that does not follow the movement of the retinal wall, between the retinal wall and retro-oil fluid was stronger in the 80% SO-filled eyes than in the 95% SO-filled eyes. Therefore, to reduce the absolute velocity gradient of the retro-oil fluid, SO close to 100% in the vitreous cavity and restricting the position of the patient more tightly to tamponade the inferior retina may be needed if the percentage of SO in the vitreous cavity is 80% compared to cases when it is 95%.

On the other hand, oil overfill can lead to increased intraocular pressure, glaucoma, corneal decompensation, and pain^13,14^. The supine position may cause some postoperative complications, such as pupillary block, anterior chamber shallowing, intraocular lens dislocation/iris capture, and SO migration into the anterior chamber. Therefore, the amount of SO in the vitreous cavity and the postoperative position should be carefully selected for each case. Yoon et al. reported that the adhesive force was transiently reduced after laser photocoagulation of the retina, but increased beyond normal and remained twice that of normal between 3 days and 4 weeks postoperatively^24^. Therefore, patients may be free of positional restrictions after postoperative day 3.

It is well known that myopia is correlated with increased risk of RRDs^25^. Julia et al. reported that the shear stress on the retinal wall increases significantly with the refractive error^26^. Since we used 3-D vitreous cavities of real patients, we were able to simulate the extent of oil-induced tamponade accounting for the irregular shape of the eye. In cases 1 and 3, which had more asymmetrically and irregularly shaped vitreous cavities, the absolute velocity gradient of the retro-oil fluid increased more extensively than in other cases. The maximum absolute velocity gradient was also higher in cases 1 and 3, especially in the horizontal and vertical saccadic eye movements, compared to other cases (Fig. 1, Supplementary files 1–6). These factors may play a role in the recurrence of inferior PVR in myopic eyes after PPV with SO tamponade. However, because the number of myopic eyes included in this study was small, further study involving more cases is needed to elucidate the distribution of increasing absolute velocity gradients in relation to eye shape in myopic eyes.

This study is subject to several limitations. First, CFD analysis may require a physical model for verification and validation, which was not performed in this study. Although the MPS method was shown to reproduce the experiment well in the case of sloshing^27–30^, which is similar to this study and suggests that our results have certain reliability, the lack of verification of the validity and reliability of the used particle method might limit the accuracy of our results. Further research will be required to establish a valid model based on postoperative intraocular conditions and the morphological characteristics of the vitreous cavity. Second, the extracted 3-D object of the vitreous cavity was a polygon without elasticity, meaning that deformation could not be considered, so changes in the position of the intraocular lens and intraocular pressure due to oil movement cannot be reproduced. Third, owing to the limited resolution of MRI, fine irregularities caused by blood vessels on the retinal surface could not be reproduced. Fourth, we did not consider the presence of air in the vitreous cavity when the oil was inserted after air displacement. However, in cases where air remains in the vitreous cavity, the air disappears early after surgery and the patient’s position should be restricted in anticipation of the space with air being replaced by retro-oil fluid. Lastly, we used the physical properties of water for the retro-oil fluid; however, these properties may differ between the two fluids.

## Conclusion

Our study is the first to analyze the oil cover rates of the inferior retina and the absolute velocity gradient of the retro-oil fluid caused by saccadic eye and head movements using the 3-D vitreous cavity model from MRI scans of real patients. We demonstrated that a vitreous cavity filled with 95% SO and the appropriate postoperative positional restrictions may improve the oil cover rate of the inferior retina and reduce the absolute velocity gradient of the retro-oil fluid in the retina. Our study is also the first to apply a particle method on analysis of intraocular fluid currents. We found that the MPS method can be used to analyze fluid currents in the vitreous cavity of complex shapes such as myopic eyes. These results may contribute to better surgical success rates for inferior PVR requiring SO tamponade for treatment and better understanding of the pathogenesis of diseases associated with an irregular shape of the vitreous cavity.

## Supplementary Information

Supplementary Video 1.

Supplementary Video 2.

Supplementary Video 3.

Supplementary Figure 1.

Supplementary Figure 2.

Supplementary Figure 3.

Supplementary Figure 4.

Supplementary Figure 5.

Supplementary Figure 6.

Supplementary Legends.

## Data Availability

The datasets generated and/or analyzed during this study are not publicly available but are available from the corresponding author on reasonable request.
