# Publisher Correction: Application of moving particle semi-implicit (MPS) method on retro-oil fluid using three-dimensional vitreous cavity models from magnetic resonance imaging

**DOI:** 10.1038/s41598-022-07282-5

**Published:** 2022-02-23

**Authors:** Makoto Gozawa, Naoki Watanabe, Kentaro Iwasaki, Yoshihiro Takamura, Masaru Inatani

**Affiliations:** 1 Department of Ophthalmology, Faculty of Medical Sciences, University of Fukui, 23-3 Shimoaizuki, Matsuoka, Eiheiji, Yoshida, Fukui 910-1193 Japan; 2 Prometech Software Inc, Round Terrace Fushimi, 3F, 17-26, Nishiki 1-chome, Naka-ku, Nagoya 460-0003 Japan

Correction to: *Scientific Reports* 10.1038/s41598-022-05886-5, published online 02 February 2022

In the original version of this Article Naoki Watanabe was incorrectly affiliated with ‘Department of Ophthalmology, Faculty of Medical Sciences, University of Fukui, 23-3 Shimoaizuki, Matsuoka, Eiheiji, Yoshida, Fukui, 910-1193, Japan’. The correct affiliation is listed below.

Prometech Software Inc, Round Terrace Fushimi, 3F, 17-26, Nishiki 1-chome, Naka-ku, Nagoya 460-0003, Japan

The original Article has been corrected.

