# Supplementary figures and images for: Application of moving particle semi-implicit (MPS) method on retro-oil fluid using three-dimensional vitreous cavity models from magnetic resonance imaging
Source: Sci Rep. 2022 Feb 2;12:1735. doi: 10.1038/s41598-022-05886-5 (PMC8810992; doi:10.1038/s41598-022-05886-5)

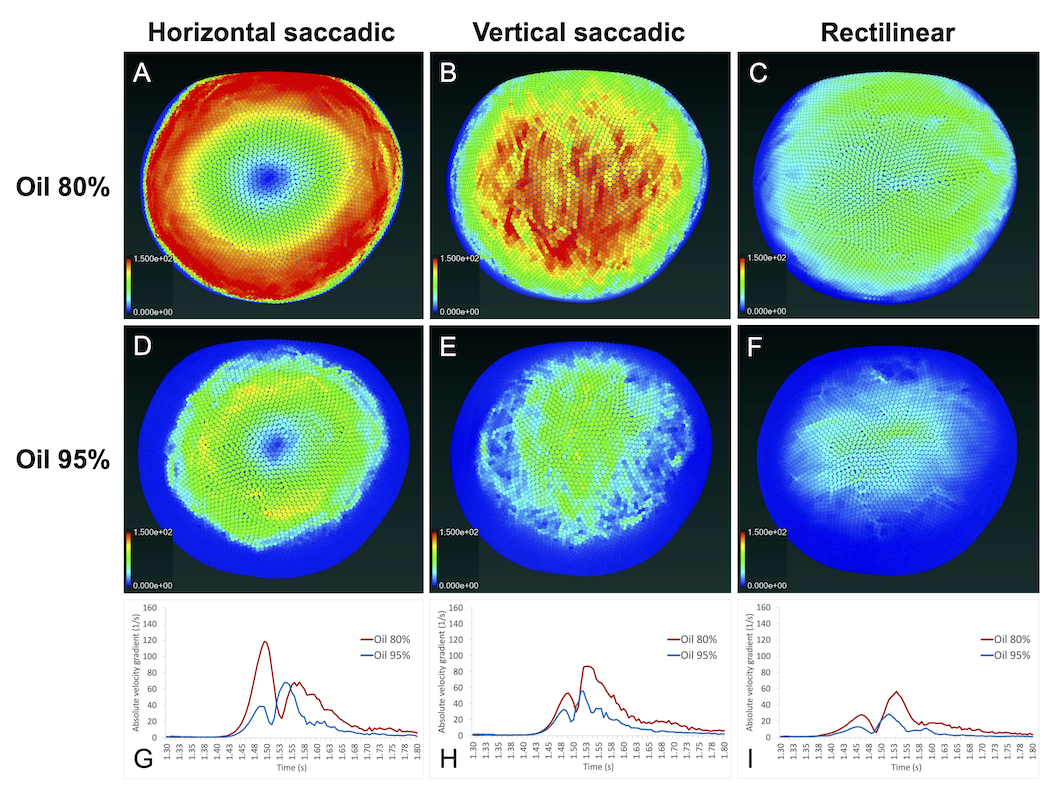

Supplement: Supplementary file 4 — Supplementary Figure 1. [file 41598_2022_5886_MOESM4_ESM.tiff]

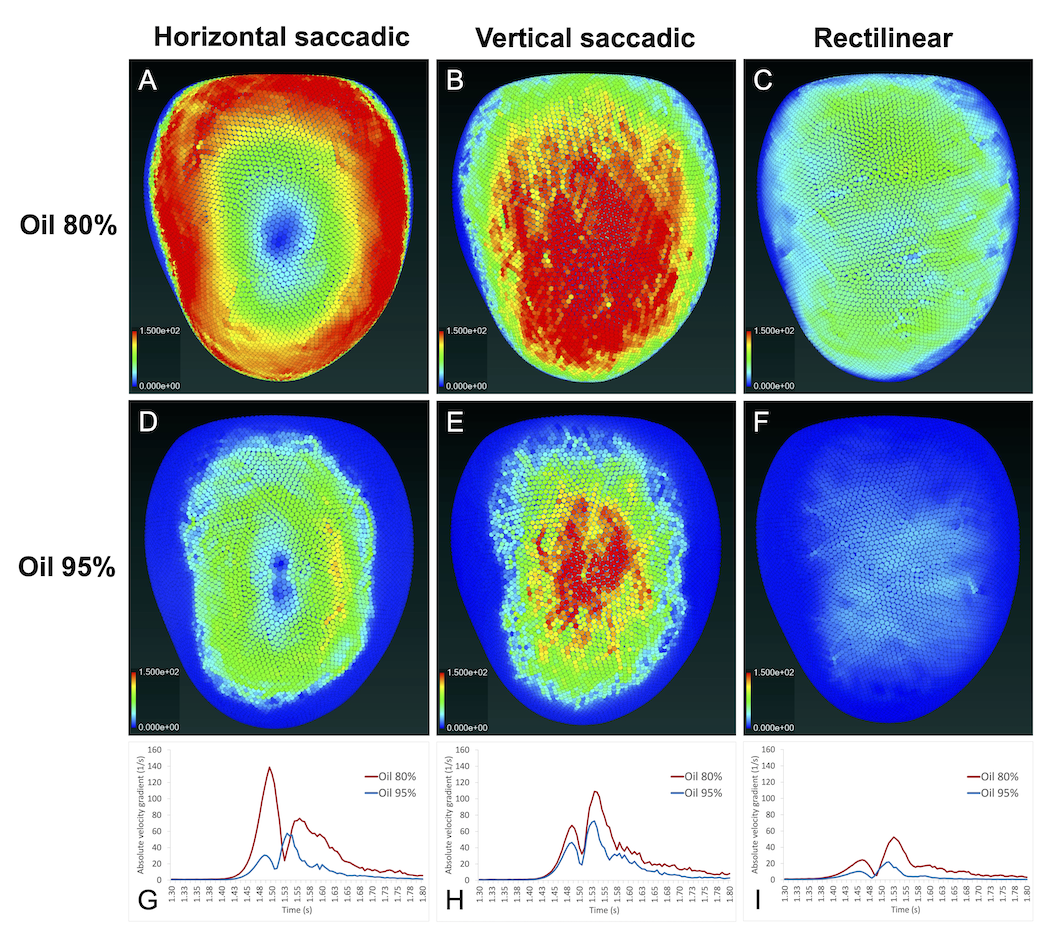

Supplement: Supplementary file 5 — Supplementary Figure 2. [file 41598_2022_5886_MOESM5_ESM.tiff]

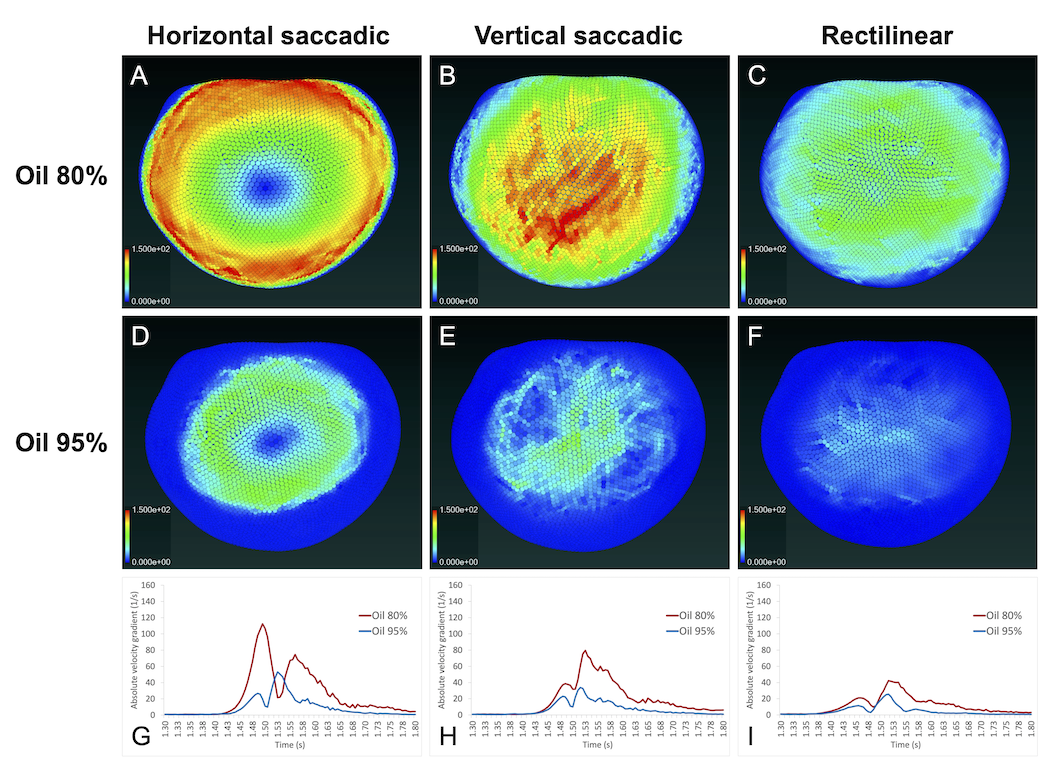

Supplement: Supplementary file 6 — Supplementary Figure 3. [file 41598_2022_5886_MOESM6_ESM.tiff]

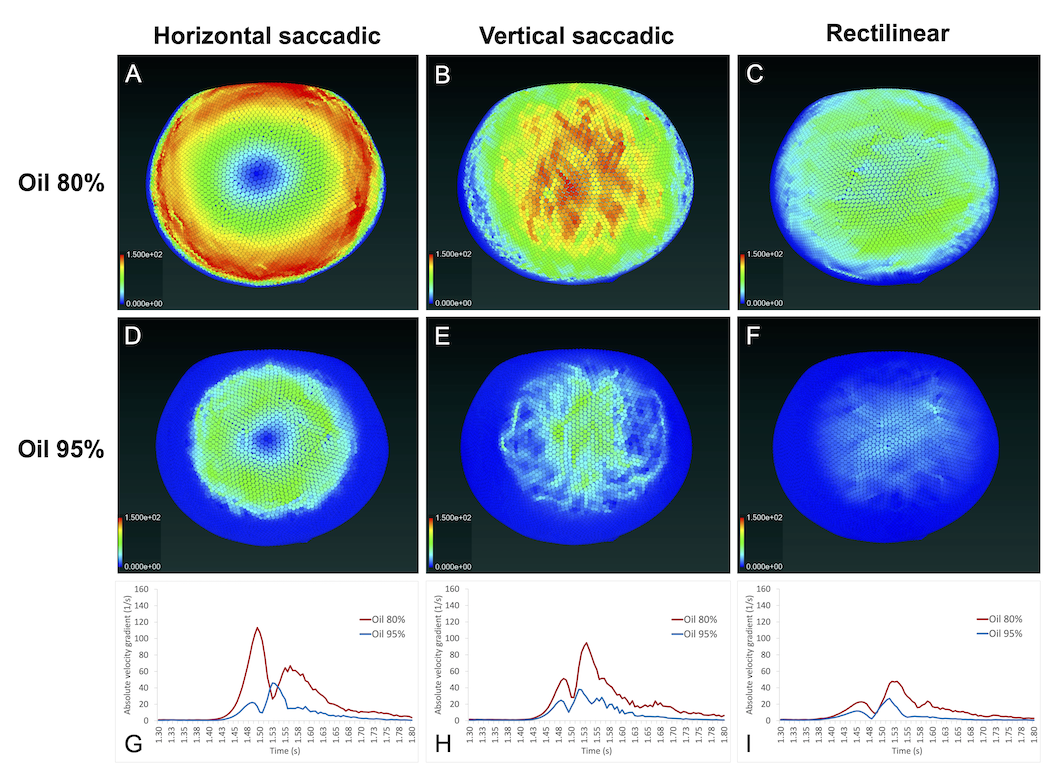

Supplement: Supplementary file 7 — Supplementary Figure 4. [file 41598_2022_5886_MOESM7_ESM.tiff]

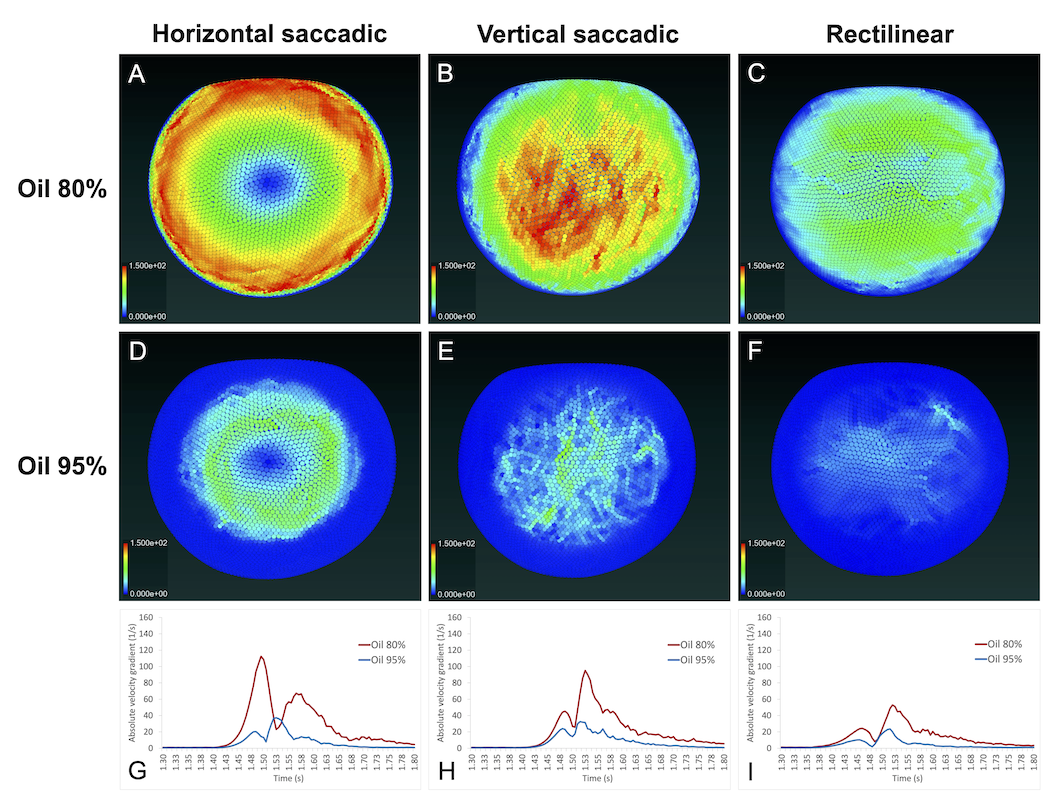

Supplement: Supplementary file 8 — Supplementary Figure 5. [file 41598_2022_5886_MOESM8_ESM.tiff]

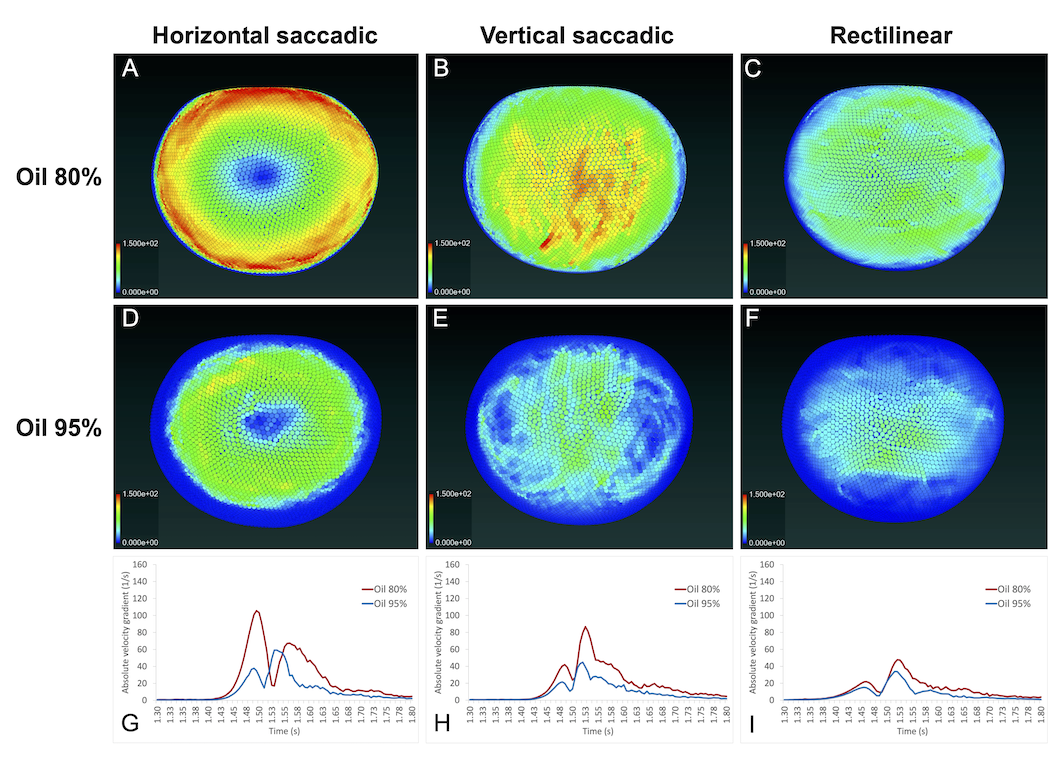

Supplement: Supplementary file 9 — Supplementary Figure 6. [file 41598_2022_5886_MOESM9_ESM.tiff]
