# Supplementary material for: Application of moving particle semi-implicit (MPS) method on retro-oil fluid using three-dimensional vitreous cavity models from magnetic resonance imaging
Source: Sci Rep. 2022 Feb 2;12:1735. doi: 10.1038/s41598-022-05886-5 (PMC8810992; doi:10.1038/s41598-022-05886-5)
Supplement: Supplementary file 10 — Supplementary Legends. [file 41598_2022_5886_MOESM10_ESM.docx]

**Supplementary file 1. Absolute velocity gradient of the silicone oil and retro-oil fluid on the retinal wall in case 2**

(A)–(F) Analysis images obtained at the maximum mean absolute velocity gradient. (G)–(I) Changes in the mean absolute velocity gradient caused by saccadic eye and rectilinear head movements.

**Supplementary file 2. Absolute velocity gradient of the silicone oil and retro-oil fluid on the retinal wall in case 3**

(A)–(F) Analysis images obtained at the maximum mean absolute velocity gradient. (G)–(I) Changes in the mean absolute velocity gradient caused by saccadic eye and rectilinear head movements.

**Supplementary file 3. Absolute velocity gradient of the silicone oil and retro-oil fluid on the retinal wall in case 4**

(A)–(F) Analysis images obtained at the maximum mean absolute velocity gradient. (G)–(I) Changes in the mean absolute velocity gradient caused by saccadic eye and rectilinear head movements.

**Supplementary file 4. Absolute velocity gradient of silicone oil and retro-oil fluid on the retinal wall in case 5**

(A)–(F) Analysis images obtained at the maximum mean absolute velocity gradient. (G)–(I) Changes in the mean absolute velocity gradient caused by saccadic eye and rectilinear head movements.

**Supplementary file 5. Absolute velocity gradient of silicone oil and retro-oil fluid on the retinal wall in case 6**

(A)–(F) Analysis images obtained at the maximum mean absolute velocity gradient. (G)–(I) Changes in the mean absolute velocity gradient caused by saccadic eye and rectilinear head movements.

**Supplementary file 6. Absolute velocity gradient of silicone oil and retro-oil fluid on the retinal wall in case 7**

(A)–(F) Analysis images obtained at the maximum mean absolute velocity gradient. (G)–(I) Changes in the mean absolute velocity gradient caused by saccadic eye and rectilinear head movements.

**Video legends**

**Video 1. Horizontal saccadic movements of the three-dimensional vitreous cavity of case 1 filled with a combination of 80% silicone oil and 20% retro-oil fluid.**

**Video 2. Vertical saccadic movements of the three-dimensional vitreous cavity of case 1 filled with a combination of 80% silicone oil and 20% retro-oil fluid.**

**Video 3. Rectilinear head movements of the three-dimensional vitreous cavity of case 1 filled with a combination of 80% silicone oil and 20% retro-oil fluid.**
